# Renewable energy and sustainable communities: Alaska's wind generator experience^†^

**DOI:** 10.3402/ijch.v72i0.21520

**Published:** 2013-08-05

**Authors:** R. Steven Konkel

**Affiliations:** Associate Professor of Environmental Health Sciences, Department of Health Sciences, University of Alaska Anchorage, Anchorage, AK, USA

**Keywords:** Alaska's wind generators, renewable energy, wind generation, Alaska energy policy

## Abstract

**Background:**

In 1984, the Alaska Department of Commerce and Economic Development (DCED) issued the State's first inventory/economic assessment of wind generators, documenting installed wind generator capacity and the economics of replacing diesel-fuel-generated electricity. Alaska's wind generation capacity had grown from hundreds of installed kilowatts to over 15.3 megawatts (MW) by January 2012.

**Method:**

This article reviews data and conclusions presented in “Alaska's Wind Energy Systems; Inventory and Economic Assessment” (1). (Alaska Department of Commerce and Economic Development, S. Konkel, 1984). It provides a foundation and baseline for understanding the development of this renewable energy source.

**Results:**

Today's technologies have evolved at an astonishing pace; a typical generator in an Alaska wind farm now is likely rated at 1.5-MW capacity, compared to the single-kilowatt (kW) machines present in 1984. Installed capacity has mushroomed, illustrated by Unalakleet's 600-kW wind farm dwarfing the original three 10-kW machines included in the 1984 inventory. Kodiak Electric had three 1.5-MW turbines installed at Pillar Mountain in 2009, with three additional turbines of 4.5-MW capacity installed in 2012. Utilities now actively plan for wind generation and compete for state funding.

**Discussion:**

State of Alaska energy policy provides the context for energy project decision-making. Substantial renewable energy fund (REF) awards – $202,000,000 to date for 227 REF projects in the first 5 cycles of funding – along with numerous energy conservation programs – are now in place. Increasing investment in wind is driven by multiple factors. Stakeholders have interests both in public policy and meeting private investment objectives. Wind generator investors should consider project economics and potential impacts of energy decisions on human health. Specifically this article considers:

- changing environmental conditions in remote Alaska villages,
- impacts associated with climate change on human health,
- progress in better understanding wind energy potential through resource assessments and new tools for detailed feasibility and project planning,
- need for comprehensive monitoring and data analysis, and
- state funding requirements and opportunity costs.

**Conclusion:**

The energy policy choices ahead for Alaska will have important implications for Arctic population health, especially for those villages whose relatively small size and remote locations make energy a key component of subsistence lifestyles and community sustainability. Wind generation can contribute to meeting renewable energy goals and is a particularly important resource for rural and remote Alaskan communities currently dependent on diesel fuel for generating electricity and heat.

Alaska's installed wind generation capacity has increased enormously over the past 3 decades. Policies developed and supported during Hon. Gov. Jay Hammond's two-term administration led to additional energy programs and investment in RD&D (research, development and demonstration) of energy efficiency, energy conservation and renewable energy technologies (1–4). Installed wind capacity as of January 1, 2012 was 15.3 MW (5). The overall capacity may more than triple as 2 new wind farm projects in the Railbelt (the Seward–Anchorage–Fairbanks corridor) add capacity on Fire Island in Cook Inlet and at Eva Creek near Healy. There are 11 turbines installed on Fire Island and Chugach Electric Association (CEA) has a contract to buy the electricity, while Golden Valley Electric Association (GVEA) is constructing a 24.6-MW wind farm at Eva Creek, 14 miles from Healy, in the Interior (6).

In 1984, the Alaska Department of Commerce and Economic Development (AK DCED) produced Alaska's first inventory and economic assessment of wind generators (1). (AK DCED, S. Konkel, 1984; see Figs. 1 and 2 for historical and current photographs). This report documented installed wind generator capacity, a method for project evaluation, and the economics of supplementing diesel-fuel-generated electricity in Alaska's rural and remote villages (Bush Alaska). Information on the economic impacts of Alaska's energy programs on rural energy use was presented at the State's annual alternative energy conferences held in the 1980s.

**Fig. 1:**
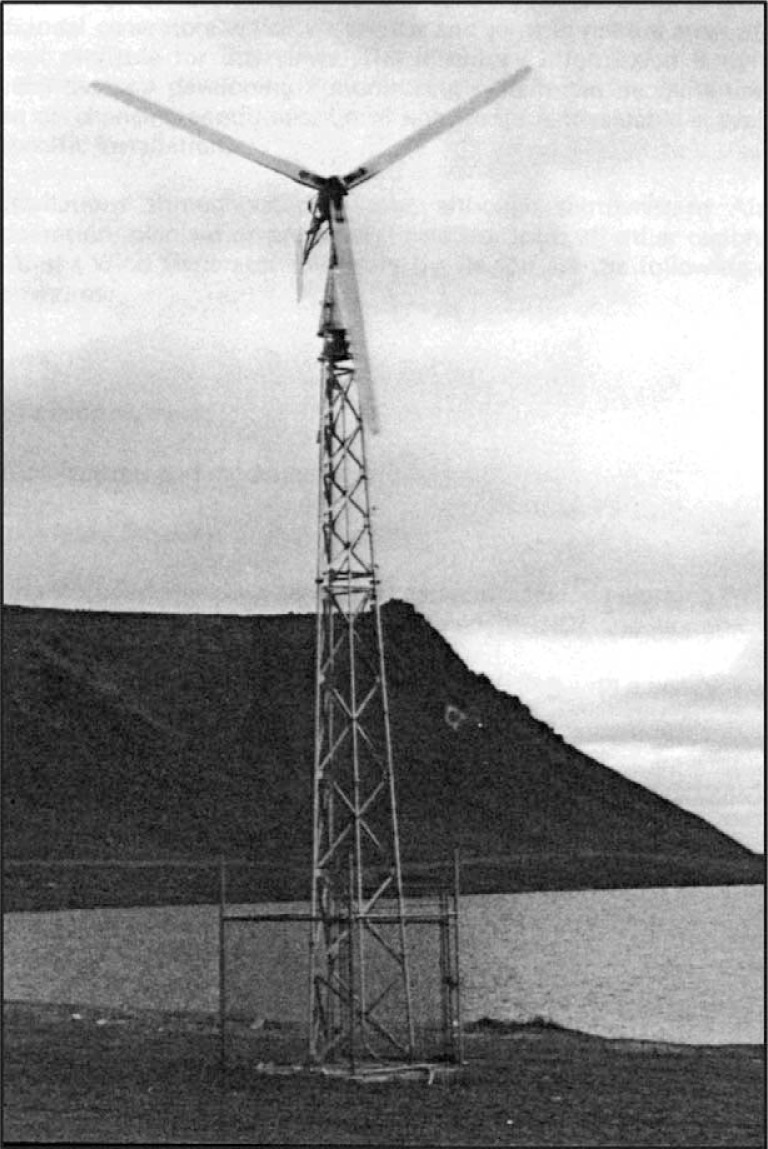
A 10-kW wind generator in Gamble on St. Lawrence Island. (Photo credit, S. Konkel, 1983)

**Fig. 2:**
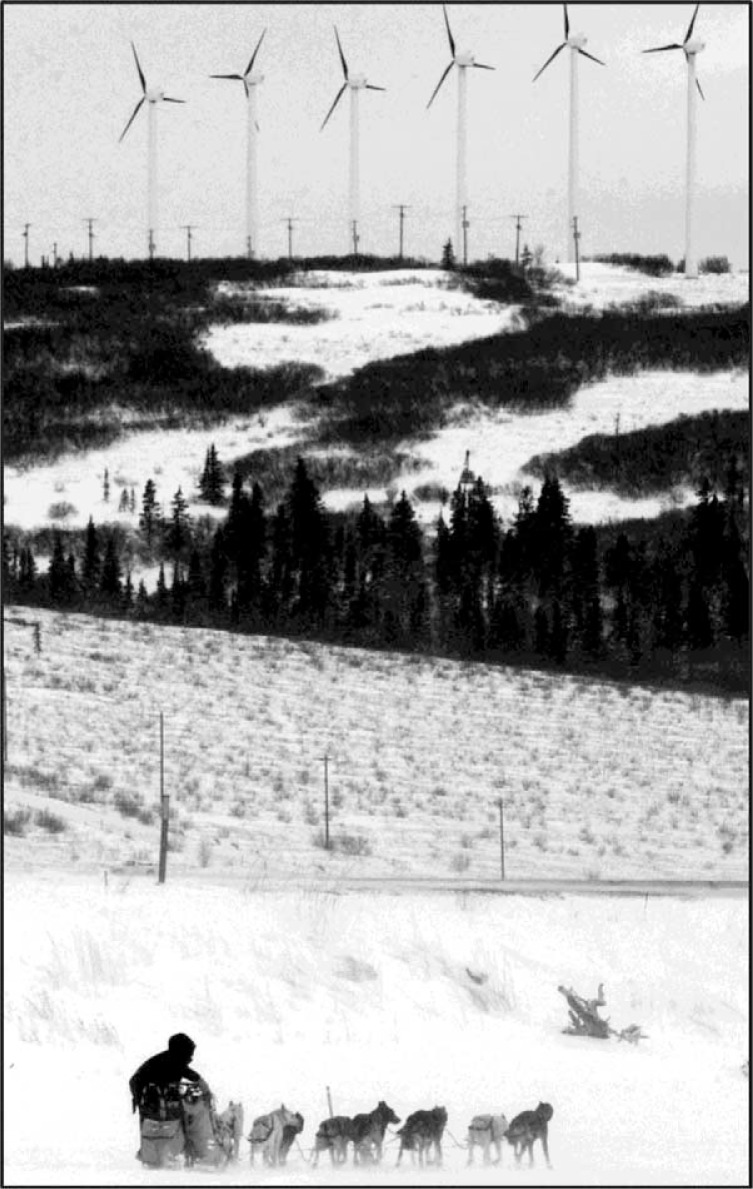
Mitch Seavey was the first musher to leave the check point at Unalakleet on Sunday in the Iditarod Trail Sled Dog Race. Note the six 100-kW wind generators on the ridge. Photo Credit: Bill Roth, Anchorage Daily News, March 13, 2013.

**Fig. 3:**
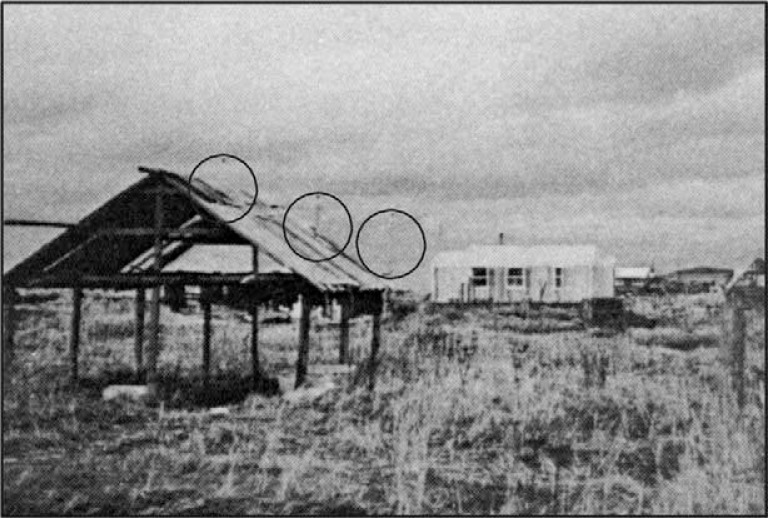
Three 10-kW wind generators were originally sited in Unalakleet in the early 1980s behind the fish drying rack in the foreground. This was one of the first utility scale wind generation systems installed in Alaska. Kotzebue Electric Association was one of the first utilities pioneering the use of wind generators in Arctic Alaska.

The Alaskan landscape has changed with new larger kilowatt (kW)/megawatt (MW) machines, new agency responsibility for renewable energy, federal energy grants and wind development conceptual guidelines (AEA). There are substantial State matching grants for financing renewable energy projects. Alaska's Renewable Energy Fund (REF), passed by the Legislature as HB 152, has spurred a level of investment where the equivalent of 6 million gallons of diesel fuel was expected to be displaced by renewables by 2013 (5). AK DCED's Division of Energy and Power Development (AK DEPD) had responsibility for R&D for wind projects in the 1980s.

Over the past 30 years, policies and conditions driving the installation of wind generators have evolved to support technology investments. Diesel fuel prices have continued to increase in the Bush due to world oil prices and increased costs for transportation to villages, with air transport being almost prohibitive in price. This is a key cost-of-living factor in Alaska's rural and remote villages. Diesel fuel costs averaged more than $6 per gallon in rural communities in 2012, compared with more than $4 per gallon in urban Alaska, according to ANTHC's Alaska Rural Utilty Collaborative (ARUC) program (7–9) (Konkel, 2007, S. Konkel, 2012).

In the Northwest Arctic Borough in particular, Alaskan villages have been experiencing changes associated with increases in average temperatures due to climate change, such as melting permafrost, freeze/thaw cycles, loss of shorefast ice, coastal erosion, and more frequent and severe storms (10). Traditional subsistence hunting patterns have also been affected. Changes in environmental conditions in Arctic Alaska suggest that robust and effective energy policy can help secure the State's future. Certain emissions from power-generating facilities can have a much greater impact on health, with carbon-based fuels presenting air quality and greenhouse gas emission challenges (11,12). Renewables have a place. In the Hammond Administration, projects and programs were evaluated using a three-part test: (a) Are the environmental impacts acceptable? (b) Do Alaskans want the project? and (c) Does the project pay for itself?

The increasing evidence of shrinking sea ice in the Arctic and the potential impact of accelerated warming in areas above the Arctic Circle suggest that reducing atmospheric emissions from carbon-based fuels is necessary for adaptation to the increasingly evident impacts of climate change (13,14). Saving diesel fuel burned in diesel generators is a popular indicator used by utilities. Polar bears, ringed and bearded seals alike have been affected by changes in environmental conditions. In 2009, Secretary of the Interior Dick Kempthorne listed the polar bear as “threatened”. In 2012, the Economist published a special report on “The Melting Arctic” highlighting the loss of summer ice and the effect of warming on arctic populations (13). Norway, Denmark, Canada, the United States and Russia are engaged in talks to protect fish stocks in common arctic waters. Certainly open oceans will have impacts on fisheries and trade, and perhaps the development of energy resources, such as oil in the Chukchi and Beaufort Seas of the Arctic Ocean (14). With these developments come trade-offs and likely changes in the prospects for sustainability of Alaska Native villages.

Today, Susitna-lower Watana is backed as a centerpiece of Alaska's energy policy (15). One example illustrating the stakes is a cost of power reference case where the State of Alaska pays “50% of the $5.0 billion (in 2008) construction cost of the Susitna–Watana hydroelectric project as a direct cash contribution” (16) (Colt, S. 2012, p. 15). This is sometimes characterized as a grant by proponents of the project or as a subsidy by critics. Regardless, consumers pay only a portion of the real costs. Subsidy programs, like Power Cost Equalization, distort prices that utilities and consumers pay; these affect the willingness to invest in projects and to realize the benefit of renewable energy project production.

Alaska has an ambitious goal to meet 50% of electricity demand through renewables by 2025, an attractive goal set in 2010 as the Alaska Legislature passed HB 306. However, the “devil” is in the details, since there are tradeoffs and opportunity costs to funding projects. Projects costing billions of dollars, like a proposed $45–65 billion natural gas pipeline from the North Slope, and the $5.2 billion (estimated cost) Susitna–lower Watana dam would have substantial price tags. Financing these “mega-projects” using State direct cash contributions has been the subject of many energy debates over the years. One alternative to spending money on projects is investing in the principal of the Permanent Fund, an account created during the Hammond Administration, where interest is distributed to Alaska residents in the form of annual dividends. Energy projects should incorporate the costs of mitigating adverse environmental impacts, which is referred to as “internalizing the externalities.”

By the end of 2012, a wind farm had been constructed on Fire Island in Cook Inlet; 33 turbine locations had been permitted and 17.6 MW of capacity installed as of February 2013. Phase one consisted of 9 wind generators located in the middle of the island along with 2 on the southeast coast. Cook Inlet Region Inc., a Native corporation, and CEA are developing the Fire Island wind project. On Kodiak Island, there are 6 wind generators; each of these has a rated capacity of 1.5 MW. Eva Creek will likely be the largest of all of the wind farm projects. GVEA has over 24.6 MW of installed capacity (with turbines rated at 2.05 MW) and this power is integrated into and regulated with other energy sources. These projects illustrate the future of the technology and the context of its integration with other electrical energy sources, such as diesel generators. Hybrid-type wind in combination with other energy systems face technical and economic challenges.

In the Seward–Anchorage–Fairbanks corridor, also known as the Railbelt, 6 cooperative utilities serve over half of the State's population. Natural gas dominates the fuels used statewide; with 57% of the electricity generated; hydroelectric projects generate 22% with diesel fuel at a substantial 15% and coal at 6% (17). Wind projects are designed to displace significant amounts of diesel fuel.

As noted, the REF has provided grants and financing for 227 projects. The Alaska Legislature appropriated $150,000,000 for the first 2 years under HB 152, then an additional $25,000,000/year. Now the program is in Cycle 6, where 85 new project proposals are being evaluated by AEA for 2013 awards. Some of the current challenges for wind generation projects include storage of energy, upgrading diesel generation systems to address efficiency in meeting electrical loads while integrating power from wind generators, and investments required for sustainable utilities in rural Alaska (18). In 2010 Alaska's wind generator installed capacity at 9MW was very small compared to other states. For example, Colorado – 1,299 MW, Washington – 2,104 MW, Oregon – 2,104 MW and California – 3,177 MW (5). (Renewable Energy Atlas, p. 23). For the purpose of comparison, the 2010 Alaska Railbelt Regional Integrated Resource Plan (RIRP) forecasted a summer peak of 668 MW and a winter peak of 869.3 MW (19). Alaska has a relatively modest demand compared to highly populated states like California. But Alaska's installed wind capacity is growing.

## Methods

The 1984 inventory and economic assessment provided a “baseline” of wind generators, installed kilowatt capacity and a forecast of capacity factors at that time. It also included a methodology for looking at the production of the machines, in terms of their potential to replace diesel fuel. Average fuel prices in 2007 in Alaskan communities were $3.25–$5.25 per gallon as documented by ARUC program (9) (Konkel, 2007, pp. 5–10).

A number of high-visibility policy and planning documents, including ISER reports, have provided insights into rural energy investment, Railbelt electricity (e.g. Susitna-lower Watana dam, possible Cook Inlet and North Slope natural gas field supply scenarios) and Alaska's current fiscal climate with declining oil revenues (16, 20–22). These provide a context for energy policy and capital appropriations.

In the discussion section, recommendations are made for addressing key issues affecting wind generation in Alaska. There may be useful insights from comparing energy policies and technologies in the 8 countries of the Circumpolar North. Alaska is somewhat unique in that due to its Constitution it is an owner state; the natural resources and minerals belong to the State's residents for the joint purposes of development and stewardship. Also, Alaska is unique in having $126 billion in petroleum wealth – with $45 billion in savings accounts derived from oil revenues and the remainder still in the ground (21) (Goldsmith, p. 1). Alaska has a long history of subsidizing capital investments in energy projects and even consumption with power cost equalization – part of the hydroelectric project investment political compromise to spread benefits statewide. Wind can make a modest MW contribution to meeting Alaska's renewable energy goal of 50% from renewables by 2025, a goal legislated in 2010.

There are laws that complement the REF. SB 220 mandated, among other things, that one-quarter of the State's buildings be retrofitted for energy conservation by 2020 and a $250 million revolving loan fund be established to finance that work. HB 306 established the renewable energy goal of 50% of the State's electricity be derived from renewable energy sources by 2025 and per capita energy use be decreased by 15% by 2020. HB 152 established the Alaska Renewable Energy Grant Fund.

This article also reflects on past Administrations’ energy planning – the author was a Policy Analyst in the Office of the Governor, Hon. Gov. Jay Hammond's second administration, where responsibilities included oversight for conservation and renewable energy program and R&D from 1980 to 1982. The AEA now has taken over the energy planning and implementation responsibilities formerly led by the DEPD and the Alaska Power Authority. The high cost of providing electricity and sustainable utilities has important policy implications for the future of Alaska villages.

## Results

To see how far we have come with the siting of wind generators, also known as WECS (wind energy conversion systems), we compare today's installed capacity with the comprehensive wind inventory and economic assessment conducted in 1984 by the Alaska Department of Commerce and Economic Development, (1) (AK DCED, Konkel, 1984). Table I shows the number of wind generators by region and the following Appendix B Alaska's Wind Energy Systems provides information on the number of wind generators in each region, and insights into their capacity, make, status and capacity factors in 1984.

**Table I:** Number of wind generators by region

Region	Number of wind generators
Northern (Arctic)	5
Northwest	27
Western	19
Southcentral	23
Southwestern	30
Southeastern	11
Interior	9
Aleutians	16
Total	140

Much has changed, perhaps on a scale unimaginable since the 1980s. Unalakleet added a 600-kW wind farm, consisting of six 100-kW wind generators in 2009. This same year Kodiak installed 3 General Electric 1.5-MW turbines; by 2012, there were 3 more planned for the Pillar Mountain wind project. In 2009, the 3 wind generators had cut Kodiak Electric Association's (KEA) diesel fuel use in half, saving 930,000 gallons during the first year of operation. KEA has estimated that the project replaced the equivalent of 3,568,395 gallons of diesel fuel from July 2009 to January 2013, representing over $10,000,000 in fuel costs in that period. Kotzebue Electric Association currently has wind generators of the following rated kW capactity: 900-kW (two), 66-kW (fifteen), 65-kW (one) and 100-kW (one); a comparison of output and capacity factors, given their production history and common location, would be insightful.

As of January 1, 2010, Alaska's installed wind generator capacity totalled 9 MW. This represents only 0.5% of the statewide electrical generation. As noted earlier, major sources of electrical generation in Alaska include natural gas at 57%, coal at 6%, diesel fuel at 15% and hydroelectric power at 22% (21) (ISER 2012, “Energizing Alaska: Electricity around the State, p. 1). Outside the Railbelt, wind generation, in concert with diesel generators may have a niche in the Bush, where wind generator production can replace significant amounts of diesel fuel. This technology also reduces the substantial externality costs of relying on oil. Technology proponents and non-governmental organizations are concerned about the impacts of increased emissions of CO_2_, global warming (exacerbated at northern latitudes), and increased impacts on permafrost and environmental conditions generally unfavourable to achieving sustainable village utilities. Although wind is intermittent, using its output for space heating or to prevent freezing of drinking water and sanitation systems has potential benefit. In short, wind generators could make an important contribution, scalable from village to village depending on wind regimes, to lessen dependence on high-cost diesel fuel and improve the reliability of critical infrastructure. This type of energy policy has benefits to a state that is more vulnerable than most to changing environmental conditions and a state that lives with perpetually high-energy prices.

Looking back since the initial installation of wind projects, one observes low production of total kilowatt-hours (kWh) per year. Installed capacity of 1 or even 2 kW to the three 10-kW machines in Unalakleet (Fig. 3) or the 20-kW machine in Nelson Lagoon pale in comparison with today's machine sizes. The Unalakleet investment was a small-scale utility project. Single household machines were most common. Tables II and III contain a variety of statistics (current as of January 1984) for wind generators in place at that time. An applied research project to update kilowatt-hour production and capacity factors over time would provide essential insight into historical production. Kilowatt-hour production from both the KEA and GVEA wind generator projects is massive compared to the historical production from these earlier wind generators.

**Table II:** Alaska's wind energy systems

Project site	ID	Size (kW)	Make	Status	kWh/year	Month of kWh data
Nelson Lagoon	1	20	Grumman	OW	21,560	14
Sand Point	2	10	Jacobs	OW	17,020	12
Naknek	3	12.5	Jacobs	W	16,900	24
Unalakleet No. 2	4	10	Jacobs	W	16,520	8
Gambell	5	10	Jacobs	W	13,800	16
Unalakleet No. 4	6	15	Jacobs	W	22,780	6
Nome	7	10	Jacobs	W	11,970	3
Hooper Bay	8	10	Jacobs	W	10,840	18
Skagway	9	10	Jacobs	W	10,370	10
Gambell	10	10	Jacobs	W	9,940	16
King Salmon	11	10	Jacobs	W	9,290	7
Kodiak	12	10	Jacobs	W	7,320	16
Pilot Station	13	10	Jacobs	W	7,200	3
Palmer	14	10	Jacobs	W	6,800	9
Unalakleet No. 3	15	10	Jacobs	W	5,460	8
Chevak	16	1.8	Enertech 1800	W	5,400	5
Kivalina	17	4	Enertech 4000	OW	4,800	3
Bethel	18	4	Enertech 4000	OW	4,400	6
Gambell	19	10	Jacobs	NW	4,120	16
Unalakleet No. 1	20	10	Jacobs	W	4,000	8
Platinum	21	1	Bergey 1000-S	W	2,875	10
Ketchikan	22	10	Jacobs	W	2,600	7
Bethel	23	1.8	Enertech 1800	NW	1,350	21
Ketchikan	24	10	Jacobs	W	1,220	5

Wind generator project statistics (Konkel, 1984, taken from Appendix B).

NW=not working; OW=occasionally working; W=working at time of the 1983 site visit.

**Table III:** Alaska's wind energy systems

Project site	Average wind speed	Capacity factor
Nelson Lagoon	15E	0.123
Sand Point	19	0.194
Naknek	13	0.154
Unalakleet No. 2	11	0.189
Gambell	18	0.158
Unalakleet No. 4	11	0.173
Nome	10	0.137
Hooper Bay	14	0.124
Skagway	10E	0.118
Gambel	18	l0.113
King Salmon	11	0.106
Kodiak	10	0.084
Pilot Station	14	0.082
Palmer	6E	0.078
Unalakleet No. 3	11	0.062
Chevak	15	0.342
Kivalina	13E	0.137
Bethel	12E	0.126
Gambell	18	0.047
Unalakleet No. 1	11	0.046
Platinum	17	0.328
Ketchikan	8E	0.030
Bethel	12E	0.086
Ketchikan	8E	0.014

Konkel, 1984, taken from Appendix B.

E=estimated from data at other sites in the same geographic region. Source for the data is the Alaskan Wind Energy Handbook. Capacity factor is calculated by dividing the estimate for annual kilowatt-hour production (kWh/year) by the rated capacity of the wind generator multiplied by 8,760 hours/year.

## Discussion – key points

- Wind turbine generator technologies can have an economic impact on the costs of generating and integrating power into utility systems in rural/remote Alaska.
- Monitoring and data analysis on production, costs and efficiency would add valuable knowledge for planning projects and improving return on investment (ROI). Rigorous analysis is required for evaluations and planning.
- The 1984 Inventory and Economic Assessment inventoried and evaluated the evidence base.
- The Alaska Renewable Energy Grant Fund has driven investment in renewables. The Legislature appropriated $202 million for 227 projects in the first five cycles of funding. Another $25 MM is available from appropriations to cover projects selected in Cycle 6, in which the AEA evaluated 85 projects. Understanding and refining this selection and appropriation process is essential. Wind generation projects or wind farms must compete with other renewable energy projects for funding.
- KEA's six 1.5-MW wind generators and Fire Island wind project's 11 turbines in Southcentral Alaska dwarf earlier investments. GVEA is planning the largest installation, over 24.6 MW for the Eva Creek project. Each utility project faces its own set of challenges, due to the intermittent nature of the wind resources, storage options/costs and costs of integration/regulation to use the wind project output.
- New funding extends the grant cycles for 10 years to 2023, with a stated goal of up to $50 MM/year. Some analysts think that $25 million a year is much more realistic given fiscal uncertainties and realities. The State of Alaska has an interesting array of options, many of which require substantial subsidies that have large opportunity costs. The renewable energy goal is an important feature of Alaska's policy, with the controversy over how to address increase in greenhouse gases being of utmost importance to Alaska as a function of its geographic location; Alaska is at the coalface, the place where changes in environmental conditions are increasingly evident to residents and decision makers.
- AEA is a lead agency, responsible for program development and issuing planning and conceptual design guidelines.
- Programmatic development and evaluation might best be furthered by:
  - incorporating program and project assessments into the next issuance (2015) of on the Renewable Energy Atlas of Alaska;
  - following and updating methodologies used in earlier wind assessments such as *Alaska's Wind Energy Systems* (1), (AK DCED, S. Konkel, 1984);
  - producing reports and case studies on best practices, integration of energy generation sources, and on lessons learned;
  - monitoring wind generators to evaluate performance and economies of scale through sustainable operation and maintenance innovations.

## Conclusion

The State of Alaska plays a key role in approving projects under a 15-year REF program. Wind turbine generators are now replacing significant amounts of diesel fuel in the rural and remote Alaska villages. Up to $50,000,000/year of renewable energy projects can be authorized by the Alaska State Legislature in the coming years. However, Alaskan budgets are under increased scrutiny due to decreased revenues; federal expenditures under sequestration in 2013 and beyond are also uncertain.

By monitoring the wind generators and collecting data on production, costs and efficiency, and careful planning to take advantage of favourable wind resources, this technology has promise to contribute to Alaska's future. Unlike technologies with carbon emissions, wind is relatively benign, with relatively minor land use and wildlife impacts compared to other energy technologies which impact air quality, generate hazardous materials with possible exposure routes affecting human health, or produce radioactive waste streams.

Looking back over the many years since wind energy systems were first installed in Alaska, we see that the people who planned and installed these small kilowatt machines were true pioneers. Much has changed over the decades; wind-generated power is now displacing expensive diesel fuel. Wind generation projects make negligible contributions to increased emissions of greenhouse gases, mostly contributing through the emissions associated with manufacturing and transportation of large mechanical components.

Given the harsh and unforgiving conditions in the Arctic, other countries in the Circumpolar North also have much to gain by adopting energy policies that favour conservation first, renewable energy sources integrated into the mix, and favoring technologies and planning that slow the impacts of climate change. A lower total diesel fuel bill in rural Alaska is not going to totally reconfigure Alaska's overall energy picture. That said, renewables can lower costs of basic utilities such as water, sanitation and electricity by saving expenditures on expensive diesel fuel. Improving energy efficiency and preventing catastrophic failures due to freezing water systems could improve the resilience of systems in places such as the Northwest Arctic Borough (Kivalina or Selawik, for instance). These measures will move us towards more sustainable communities throughout rural Alaska.

The State of Alaska should have a rural energy policy where renewables are prominent and the people of Alaska benefit most from the development of its natural resources. The challenge is to create a more energy-efficient future. We now have options driven by design and technology that were hard if not impossible to imagine in the 1980s. Technology alone cannot solve all problems. We have learned from the statewide experience with providing clean drinking water and sanitation utilities that whether capital costs are sufficiently subsidized or not, keys to success include sustainable operation and maintenance and the capacity of Alaskan villages to manage their utilities.

In conclusion, wind generators have a place in the State's current and future energy policy choices. A good strategy is to internalize the external costs of energy development and maximize community benefits. A road map on how to achieve the potential wind generation contribution would be very useful for both investors and communities. The choices ahead for Alaska will have important implications for Arctic population health, especially for those villages whose relatively small size and remote locations make energy a key component of subsistence lifestyles and community sustainability.
